# Gaining ecological insight on dietary allocation among horseshoe bats through molecular primer combination

**DOI:** 10.1371/journal.pone.0220081

**Published:** 2019-07-24

**Authors:** Miren Aldasoro, Inazio Garin, Nerea Vallejo, Unai Baroja, Aitor Arrizabalaga-Escudero, Urtzi Goiti, Joxerra Aihartza

**Affiliations:** Department of Zoology and Animal Cell Biology, University of the Basque Country UPV/EHU, Leioa, The Basque Country; Tierarztliche Hochschule Hannover, GERMANY

## Abstract

Knowledge on the trophic interactions among predators and their prey is important in order to understand ecology and behaviour of animals. Traditionally studies on the diet composition of insectivorous bats have been based on the morphological identification of prey remains, but the accuracy of the results has been hampered due to methodological limitations. Lately, the DNA metabarcoding and High Throughput Sequencing (HTS) techniques have changed the scene since they allows prey identification to the species level, ultimately giving more precision to the results. Nevertheless, the use of one single primer set to amplify faecal DNA produces biases in the assessed dietary composition. Three horseshoe bats overlap extensively in their distribution range in Europe: *Rhinolophus euryale*, *R*. *hipposideros* and *R*. *ferrumequinum*. In order to achieve the deepest insight on their prey list we combined two different primers. Results showed that the used primers were complementary at the order and species levels, only 22 out of 135 prey species being amplified by both. The most frequent prey of *R*. *hipposideros* belonged to Diptera and Lepidoptera, to Lepidoptera in *R*. *euryale*, and Lepidoptera, Diptera and Coleoptera in *R*. *ferrumequinum*. The three bats show significant resource partitioning, since their trophic niche overlap is not higher than 34%. Our results confirm the importance of combining complementary primers to describe the diet of generalist insectivorous bats with amplicon metabarcoding techniques. Overall, each primer set showed a subset of the prey composition, with a small portion of the total prey being identified by both of them. Therefore, each primer presented a different picture of the niche overlap among the three horseshoe bats due to their taxonomic affinity.

## Introduction

Traditionally, diets of free-ranging animals have been determined by direct observation of feeding bouts or food remains, and by microhistological inspection of gut contents or faeces. Even if these approaches have provided much of the currently available dietary information on wildlife, they have some limitations: either cannot be applied to elusive animals [1], are limited by ethical reasons [2,3], or the results heavily depend upon the researchers’ skills [4–6] and the remains left by preys (reviewed in [7]). In order to overcome these difficulties, researchers have innovatively adopted a wide array of molecular approaches with varying success [1, 8–12]. Nonetheless, they have not been capable of determining the diets’ components at the species level.

Conversely, DNA based dietary studies allow the examination of the range and diversity of prey taken by generalist predators/consumers [1, 13], the identification of bulk samples even within highly degraded samples such as faeces, gut contents or regurgitates [13], the processing of DNA from many different consumed species [14] and, using non-invasive procedures, also the diet characterization of elusive species [15, 16]. Consequently, DNA metabarcoding and High Throughput Sequencing (HTS) techniques have recently become common-use in dietary studies (e.g. [16–25]), offering great detection ability and identification of consumed prey and plants to the species level. However, achieved results may be biased due to the sequencing technology [26], the genetic marker choice, the performance of the primers’ and PCR amplification, and laboratory workflows or bioinformatic analyses (e.g. [27–29]).

Cytochrome oxidase gene subunit I (COI) has become the most commonly used marker region in DNA metabarcoding diet studies (e.g. [19,24]) because it has the most extensive information in genomic databases (BOLD, GenBank). Besides, it includes very short fragments of DNA–"mini-barcodes” (representative fragments of COI)–easily recoverable from degraded samples (e.g. [24, 30–32]). Amplification of such fragments is currently carried out using different primer sets with varying success. Most studies on the trophic ecology of insectivorous bats have relied on the primers proposed by Zeale [19] (e.g. [17, 33–40], but see [41, 42]). The Zeale primers have proven to be highly successful to amplify DNA from Diptera and Lepidoptera [43, 44], and therefore have been widely used. Nevertheless, primer choice is a critical step in metabarcoding studies as the resulting list of prey may be unwittingly biased [17] due to the particular taxonomic affinity of the selected primers (e.g. [28, 29, 45]).

Alberdi et al. [45] highlighted that the use of multiple primers targeting the same taxonomic group reduces the effect of each primer sets’ biases and increments the taxonomic coverage, obtaining a more complete view of the diet of the predator. Accordingly, Esnaola et al. [46] showed that five primer sets targeting sections of varying lengths within the COI region performed differently when amplifying faecal DNA of the generalist insectivorous Pyrenean desman *Galemys pyrenaicus*. Even if most of the primers used were able to identify the most common arthropod prey taxa consumed, the differences regarding less abundant prey groups were considerable, and hence the diet composition depended on the chosen primer sets. Esnaola et al. [46] found that the combination of two primer sets was the most successful, namely the Zeale primers mentioned above [24] and a second primer set modified following Gillet et al. [31], targeting a shorter 133 bp mini-COI sequence. These two primer sets have different length and degeneration levels, and allegedly best reveal the prey range of generalist predators [46].

We aim to apply the aforementioned pair of primer sets to the molecular diet analysis of an ensemble of horseshoe bats (family Rhinolophidae) composed by *Rhinolophus hipposideros* (Bechstein, 1800), *R*. *ferrumequinum* (Schreber, 1774) and *R*. *euryale* Blasius, 1853. These three species have the greatest distribution range and broadest overlap in Europe. So far, the diet of *R*. *hipposideros* and *R*. *ferrumequinum* have been analysed using only Gillet primers by Galan et al. [42], while that of *R*. *euryale* has been characterized either with Zeale primers [33,34] or with Gillet primers [42]. Based on those studies we expect that *R*. *euryale* will mainly prey upon Lepidoptera, *R*. *ferrumequinum* upon Lepidoptera, Diptera and Coleoptera and finally *R*. *hipposideros* upon Lepidoptera, Diptera and Neuroptera. In accordance with previous research [27,46], we expect that the results of each primer set will be different and complementary, coming with a more precise view of their diet, since the identification of prey species will be primer-dependent. Secondly, we want to evaluate the combination of the aforementioned two primers sets to characterize the diet overlap of the three horseshoe bat species, which show varying preferences for moths. Andreas et al. [47] studied the niche partitioning of the aforementioned horseshoe bats based on microscopic identification and showed that their trophic niche overlap was considerably low. Thus, we aim to see how the results on the overlap of their trophic niches reflect the choice of the primer set.

## Materials and methods

### Study area

The study was carried out in Karrantza and Lea-Artibai Valleys (Basque Country, Northern Iberian Peninsula). Karrantza is a hilly valley with elevations of 200–855 m a.s.l. (30T 46968E, 478950N) where the prevailing landscape consists of a mosaic of small meadows and pastures, dedicated to dairy cattle breeding, surrounded by an important hedgerow network consisting mainly of shrubs and deciduous trees. Lea-Artibai Valley is also a hilly and steep valley with elevations ranging ca 40–700 ma.s.l. (30T 53647E, 479442N), where prevailing plantations of *P*. *radiata*–and less frequently *E*. *globulus*–are interspersed with small farming patches and small deciduous and holm oak woodland patches. Limestone massifs that provide abundant natural cavities surround both valleys, characterized by Atlantic temperate oceanic climate, where rainfall occurs throughout the year (annual mean 1400mm) [33].

### Sample collection

Sampling was carried out during the breeding season, in July 2012. Within each sampling area (Karrantza and Lea-Artibai), each bat species was sampled in a different capture site. There were three roosts in Karrantza–one for each species–and two roosts in Aulesti–one used by *R*. *euryale* and *R*. *ferrumequinum*, and another one by *R*. *hipposideros*. Bats were captured with a 2 × 2 m harp trap [48] located in the entrance of the colony roosts from 00:30 a.m. onwards, as bats returned to the caves after foraging. Each captured bat was held individually in a clean cloth bag until it defecated (a maximum of 40–90 min). Each bag was used only once to avoid cross-contamination of faecal samples. Faecal material collected from each individual bat was frozen within 6 h since collection time. Bats were immediately released into the cave after handling. Considering both capture sites altogether, 24 *R*. *ferrumequinum*, 31 *R*. *hipposideros* and 18 *R*. *euryale* individuals were sampled. Individual bats were considered as sample units [49].

### Ethics statement

Capture and handling protocols followed published guidelines for treatment of animals in research and teaching [50] and were approved by the Ethics Committee at the University of the Basque Country (Ref. CEBA/219/2012/GARIN ATORRASAGASTI). Captures were performed under license from the Department of the Environment of the Regional Council of Biscay (Permit numbers G13 1061; G13 1064 and G13 1066).

## DNA extraction, PCR amplification, library preparation and sequencing

Individual faecal samples of 10–40 mg were used for DNA extraction with the DNeasy PowerSoil Kit (Qiagen, Valencia, CA), following the manufacturer steps. Extracted DNA was PCR-amplified twice using to different primer sets, targeting different mini-COI segments of the mitochondrial DNA cytochrome c oxidase subunit I barcode region (COI): Zeale primers (ZBJ-ArtF1c and ZBJ-ArtR2c) [24] were used to amplify a 157 bp section, and Gillet primers (modified LepF1 and EPT-long-univR, following [31] to amplify another 133bp section. Both amplifications were performed using QIAGEN Multiplex PCR Kit (Qiagen Iberia, S.L. Madrid) in 25 μl PCR reactions. Each reaction contained 2.5 μl Buffer 10X, 1.5 μl MgCl_2_ 50mM, 0.5 μl nNTPs 25mM and 0.125 μl of taq polymerase. In the case of Zeale primers, 0.6 μl of each primer (forward and reverse), 17.175 μl deionised water and 2 μl sample DNA were added. With Gillet primers, 0.75 μl of each primer, 14.875 μl deionised water and 4 μl sample DNA were added. Each primer set had its own PCR program, modified from the reference to the used reactive. Thermocycler conditions for Zeale primers were: 95°C– 15 min; 50 cycles of 94°C– 30 sec, 52°C– 30 sec, 72°C– 30 sec; 72°C– 6 min (modified from [51]). For Gillet primers we used: 95°C– 15 min; 40 cycles of 94°C– 30 sec, 45°C– 45 sec, 72°C– 30 sec; 72°C– 10 min [31]. For the library preparation, each sample was tagged with a unique combination of Multiplex Identifier primers (MID) [52]. PCR outputs were sequenced by Ion Torrent sequencing platform, one run making above one million reads.

### Bioinformatic analyses

Quality control, sequence pre-processing and collapsing of identical sequences into a single sequence were performed using CUTADAPT [53] and USEARCH [54]. Clustering of sequences into Operational Taxonomic Units (OTU) was carried out with the UPARSE-OTU [55] algorithm in USEARCH, at a 97% similarity threshold using the–*cluster_otus* command. OTUs were normalized in order to avoid disparities in sample reads and the ones with less than 1% frequency were filtered with USEARCH’s–*otutab_norm* and–*otutab_trim* commands.

The taxonomic assignment of each OTU was performed by comparing the representative sequence of each OTU against reference sequences in the Barcode of Life Database (BOLD; www.boldsystems.org/) using BLAST (http://blast.ncbi.nlm.nih.gov/Blast.cgi) and GenBank (http://www.ncbi.nlm.nih.gov), following the identification criteria of Clare et al. [25]. The distribution range of each species was checked in order to verify that it encompasses our study area. Species level assignments were performed when query sequences matched reference sequences above 98% similarity and 75% overlap [25]. When query sequences matched more than one species in the database, the hit with the longest alignment length was selected. Besides, as a rule, only hits with e-value below 1e-20 were accepted [56] to make sure that the match did not occur by chance. Primer outputs were also tested to see whether any of the OTUs built from them could also identify the predators themselves.

### Data analysis

To study the effect of primers on the species composition observed in the diet, we performed a permutational multivariate analysis of variance (PERMANOVA) using *adonis* with 999 random permutations in vegan 2.5–1 package [57] for R version 3.3.2 [58]. First of all, we measured the difference among colonies/sampling sites and, as it was not significant, it was no longer considered. Then we used primer set and bat species as predictor variables and the number of occurrences of prey as response variable. Jaccard’s distance measure was used to calculate dissimilarities between samples. We performed NMDS in vegan 2.5–1 package for R to visualize dissimilarities in species composition among samples. The percentage of occurrence (POO) of a given prey taxon refers to the percentage obtained with the number of occurrences of each taxon when compared with the total number of occurrences of all taxa and the frequency of occurrence (FOO) to the number of bat individuals where each taxon was found compared with the sample size [59].

Pianka’s [60] measure of niche overlap has been carried out to compare the interspecific resource partitioning of the species. For the comparison of the diet of the three bats *adonis* analyses were performed and for the pairwise analysis we used *pairwise*.*perm*.*manova* from RVAideMemoire 0.9–72 package [61].

## Results

### Diet of horseshoe bats

We successfully extracted and amplified DNA from faeces of 24 *R*. *ferrumequinum* individuals, 18 *R*. *euryale* and 31 *R*. *hipposideros*, obtaining above one million sequence reads (Table 1). 309 OTUs were then built and 135 of them were assigned to potential prey species consumed by bats.

**Table 1:** Results obtained in the different steps of bioinformatic analyses with each of the primer sets.

	ZEALE	GILLET	TOTAL
Sequence reads	112191	1003689	1115880
Primary OTUs	179	130	309
Identified OTUs	122 (68%)	69 (53%)	191
Potential taxa	112	58 (61)*	147 (150)*
Identified species	101	54 (57)*	135(138)*
Occurrences of identified sp.	350	278 (294)*	628 (644)*

“Taxa” are the sum of OTUs identified up to species and genus level. (Sequence reads: Total of reads generated from the sequencing; Primary OTUs: Total of built OTUs; Identified OTUs: Number of OTUs which have been identified in the databases with the established similarity and overlap levels; Potential prey taxa: Total number of taxa identified up to genus or species level; Potential prey species: Total number of identified species; Occurrences of potential prey: Total number of occurrences of the identified OTUs.)

*: The number in brackets belongs to the total species number identified in Gillet’s samples, and the previous one to the potential prey species (i.e., excluding those considered environmental pollution).

We identified 62 prey species of *R*. *ferrumequinum*: 34 lepidopterans, 17 dipterans, 7 coleopterans, 2 neuropterans and 1 trichopteran. Lepidoptera and Diptera were the most frequently consumed, followed by Coleoptera (Tables A, B and C in S1 Supporting Information File). Among the most frequently consumed species *Pharmacis fusconebulosa* (Hepialidae; FOO = 46%) prevailed among Lepidoptera and *Rhipidia maculata* (Limoniidae; FOO = 71%) and *Tipula maxima* (Tipulidae; FOO = 29%) among Diptera. Within Coleoptera, the most consumed were the elaterid *Stenagostus rhombeus* (FOO = 46%) and the cerambycids *Arhopalus rusticus* and *Prionus coriarius* (FOO = 42% and 17% respectively), completed with scarabeids *Aphodius* sp. and *Serica brunnea* (FOO = 29% and 17% respectively).

Out of the 81 prey species identified in faeces of *R*. *euryale*, 61 were lepidopterans, 10 dipterans, 5 neuropterans, 2 ephemeropterans, 1 trichopteran, 1 hemipteran and 1 hymenopteran. Lepidoptera was the most frequently consumed order followed by Diptera (Tables A, B and C in S1 Supporting Information File), although the FOO of most of them was less than 20%. The exceptions were the noctuids *Capsula sparganii*, *Cosmia trapezina* and *Lycophotia porphyrea* (FOO > 28%), the geometrid *Idaea biselata* (FOO = 56%) and the limonid *Austrolimnophila ochracea* (FOO = 94%).

Finally, 73 prey species were identified for *R*. *hipposideros*, including 33 lepidopterans, 28 dipterans, 3 hemipterans, 3 neuropterans, 2 coleopterans, 1 hymenopteran, 1 trichopteran, 1 spider and 1 psocopteran. Among them, Diptera were the most frequently consumed, followed in descending order by Lepidoptera, Ephemeroptera, Trichoptera and Neuroptera (Tables A, B and C in S1 Supporting Information File). Among Diptera, lesser horseshoe bats mostly preyed upon limonids *Austrolimnophila ochracea* (FOO = 100%), *Rhipidia maculata* (FOO = 58%), *Neolimonia dumetorum* (FOO = 52%), *Limonia nubeculosa* (FOO = 29%) and *Dicranomyia modesta* (FOO = 26%), and the tipulid *Tipula helvola* (FOO = 55%). They also consistently preyed upon the neuropterans *Hemerobious humulinus* and *Wesmaelius nervosus* (FOO > 19%). Noteworthy, the occurrence of most moth species was below 3 with a maximum of 8 occurrences of the autostichid *Anania hortulata*.

As a whole, 12 prey species have been identified in the faeces of the three predators: 6 were lepidopterans (*Acronicta rumicis*, *Cyclophora punctaria*, *Idaea degeneraria*, *Anaplectoides prasina*, *Noctua sp*. and *Udea ferrugalis*), 5 dipterans (*Rhipidia maculata*, *Austrolimnophila ochracea*, *Limonia nubeculosa*, *Neolimonia dumetorum* and *Tipula helvola*) and one neuropteran (*Hemerobius humulinus*).

### Performance of primers

Gillet primers yielded the highest numbers of reads, whereas Zeale ones got the highest numbers of either primary OTUs, positively identified OTUs, occurrences of prey and prey species identified (Table 1). Moreover, some of the OTUs built from Gillet primers were identified as belonging to algae and mammal species (4.41% of the total taxa), and so they must be considered as environmental pollution instead of "potential prey" consumed by bats.

**Fig 1:**
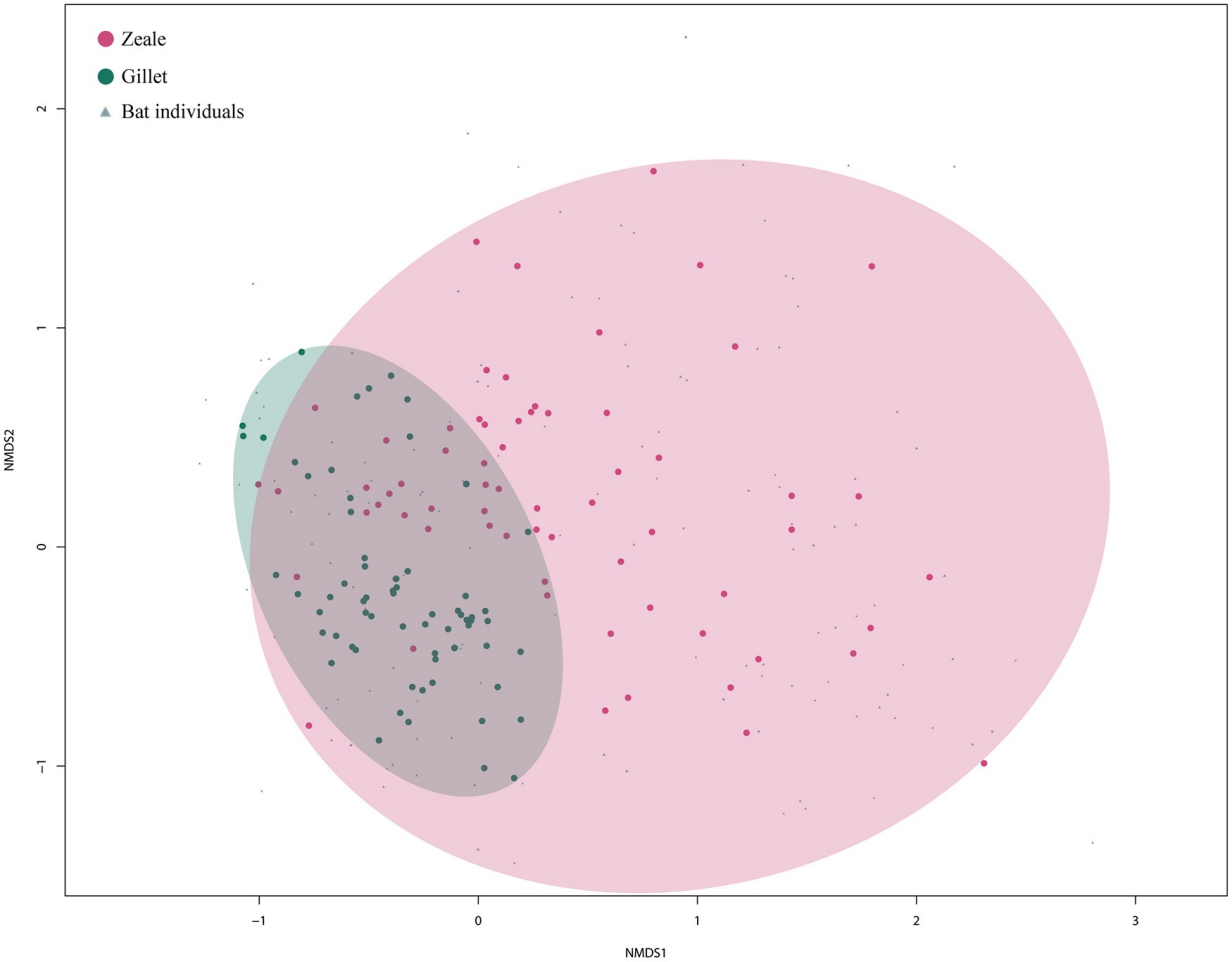
NMDS ordination of samples. Stress = 0.1997; k = 2; non-metric fit R^2^ = 0.96. Dots represent prey species and colours different primer sets (Red: Zeale; Green: Gillet). More distant dots indicate more different prey composition of samples. Individual bat samples are represented as grey triangles.

We first tested that there was not significant geographical effect of the two sampling sites in the diet (F_(1,68)_ = 1.031; R^2^ = 0.132; p = 0.37). Therefore, the location variable was not considered in further analyses. The difference between species diets is significant for the whole data set (F_(2,135)_ = 8.277; R^2^ = 0.092; p = 0.001), but also for the results obtained with each of the primer sets by their own (Gillet:F_(2,70)_ = 10.466; R^2^ = 0.230; p = 0.001; Zeale: F_(2,65)_ = 4,772; R^2^ = 0.128; p = 0.001). The primer choice significantly affected the resulting diet composition (F_(1,135)_ = 14.438; R^2^ = 0.082; p = 0.001; Fig 1). Consequently, the sum of the partial results enlarged the entire prey species list. Of the total species identified as potential prey 40% have been identified with Gillet’s set and 74.8% with Zeale’s, i.e., only 21 out of the 135 (15.5%) potential prey species have been amplified by both primer sets. Anyway, we can see that both primer and bat species affect the list of consumed prey, with a slightly higher explanation of the variation in the case of the bat species. The interaction of primer and species also shows a significant difference among the results, even if it explains less variation than primers and species on their own (F_(2,135)_ = 4.841; R^2^ = 0.055; p = 0.001). The complete lists of potential prey species identified with each primer set is are included in Tables A, B and C; sequences of all the OTUs built are available in Table D, all of them in S1 Supporting Information File.

**Fig 2:**
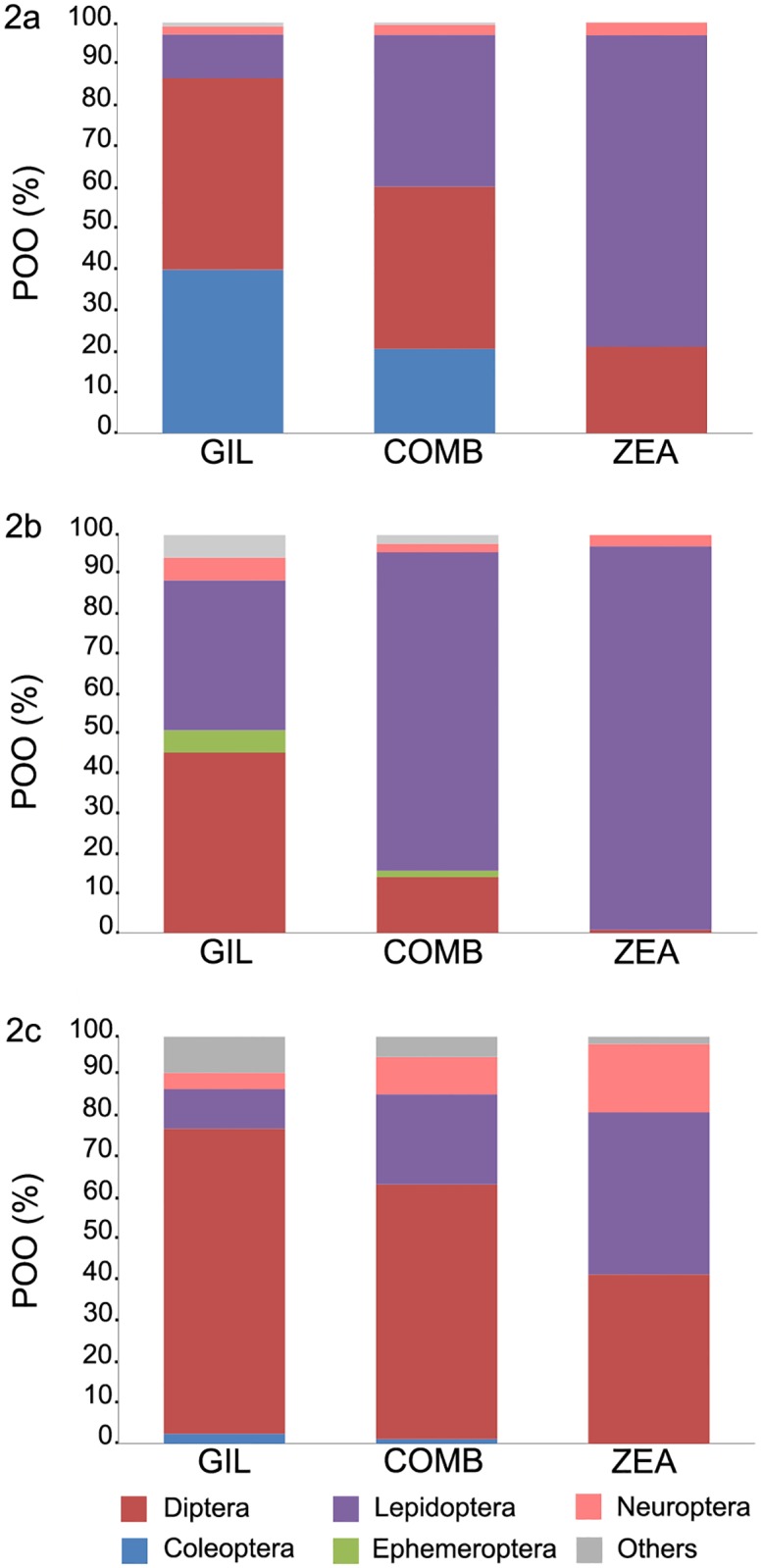
Results of the three bats’ diet obtained with each primer and combining both primers. Results are represented as percentages of occurrences (POO) (2a: *R*. *ferrumequinum*, 2b: *R*. *euryale*, 2c: *R*. *hipposideros*). “Others” comprise the orders with lesser frequencies: Araneae, hemiptera, hymenoptera, psocoptera and trichoptera. GIL: Gillet; ZEA: Zeale; COMB: Combination of both primer sets.

There were big qualitative and quantitative differences among primers at a broader taxonomic level as well (F_(1,135)_ = 61.157; R^2^ = 0.239; p = 0.001) (Fig 2, Tables 2 and 3, and Table A, in S1 Supporting Information File). Thus, Zeale primers were able to identify five orders of potential prey–namely Lepidoptera, Diptera, Neuroptera, Hemiptera and Psocoptera–whereas OTUs yielded from Gillet primers were assigned to species of fifteen orders: namely Lepidoptera, Diptera, Coleoptera, Trichoptera, Neuroptera, Ephemeroptera, Hemiptera, Hymenoptera and Araneae for prey species, as well as Mucorales, Artiodactyla, Primates and Chiroptera for environmental DNA. Besides, Zeale primers yielded more occurrences than Gillet ones (Table 1). The three predator species were identified with Gillet primers in all samples but in 2 *R*. *ferrumequinum*.

**Table 2:** Species identified in faeces and their occurrences with each primer set (Zeale’s and Gillet’s), and combining results, arranged by prey orders.

ORDER	ZEALE		GILLET		COMBINED	
	Occur. ^a^	Sp. ^b^	Occur. ^a^	Sp. ^b^	Occur. ^a^	Sp. ^b^
Araneae	0	0	2	1	2	1
Coleoptera	0	0	44	10	44	10
Diptera	68	19	165	37	269	55
Ephemeroptera	0	0	3	2	3	2
Hemiptera	1	1	5	3	6	4
Hymenoptera	0	0	3	2	3	2
Lepidoptera	256	119	43	17	281	128
Neuroptera	26	6	10	5	33	10
Psocoptera	1	1	0	0	1	1
Trichoptera	0	0	5	2	6	3

^a^Number of occurrences

^b^Species amount

**Table 3:** Main orders of prey consumed identified in faeces of the three species of horseshoe bats.

ORDER	*R*. *ferrumequinum*		*R*. *euryale*		*R*. *hipposideros*	
	FOO^a^	Sp.^b^	FOO^a^	Sp.^b^	FOO^a^	Sp.^b^
Araneae	0,00	0	0,00	0	6,45	1
Coleoptera	70,83	8	0,00	0	9,68	2
Diptera	95,83	17	94,44	10	100,00	28
Ephemeroptera	0,00	0	16,67	2	0,00	0
Hemiptera	4,17	0	11,11	1	12,90	3
Hymenoptera	0,00	0	5,56	1	6,45	1
Lepidoptera	79,17	34	100,00	61	70,97	33
Neuroptera	20,83	2	22,22	5	48,39	3
Psocoptera	0,00	0	0,00	0	3,23	1
Trichoptera	4,17	1	5,56	1	12,90	1

^a^Frequency of Ocurrence of each order

^b^Species amount

The interspecific overlap of the diet obtained with Zeale primer sets is not significantly different than the expected by chance (O_jk_ = 0.18, P = 0.076). *R*. *ferrumequinum* and *R*. *hipposideros* show the highest overlap (O_jk_ = 0.34), followed by *R*. *euryale* and *R*. *ferrumequinum* (O_jk_ = 0.12), and *R*. *euryale* and *R*. *hipposideros* (O_jk_ = 0.08). On the contrary, the overlap based on Gillet primers was significantly higher than expected by chance (O_jk_ = 0.50, P = 0.002) with the least overlap between *R*. *euryale* and *R*. *ferrumequinum* (O_jk_ = 0.25) and the highest overlap between the other two species pairs (*R*. *euryale—R*. *hipposideros*: O_jk_ = 0.74; *R*. *ferrumequinum—R*. *hipposideros*: O_jk_ = 0.52). In any case, the effect size is still generally large and the p-value generally small. Similarly, when results of both primer sets are combined the overlap was higher than expected by chance (O_jk_ = 0.34, P = 0.001). Again, the least overlap was showed by *R*. *euryale* and *R*. *ferrumequinum* (O_jk_ = 0.15), while the other two couples show a higher overlap (*R*. *euryale—R*. *hipposideros*: O_jk_ = 0.38; *R*. *ferrumequinum—R*. *hipposideros*: O_jk_ = 0.50).

## Discussion

Our results confirm the relevance of combining complementary primers to describe the diet of generalist insectivorous bats with amplicon metabarcoding techniques. In general, each pair of primers revealed a subset of the prey composition, with a small fraction of the species being detected by both of them. As a result, the interplay between the primer taxonomic affinity and dietary composition of the bat affected the niche overlap among the three horseshoe bats pictured by each primer.

Due to their more generalist character [31], Gillet primers amplify and identify a higher number of different orders, showing a more diverse diet composition. This generalist character, though, doesn’t cover a full representation or important prey orders such as Lepidoptera. Moreover, their high amplification success comes with the impossibility of identifying a substantial fraction of the amplified DNA (Table 1). On the contrary, the higher selectivity of the Zeale primer set for Lepidoptera and some Diptera [43, 44] might elicit the underestimatimation of other groups of consumed prey, such as Coleoptera, Ephemeroptera, Hymenoptera or Orthoptera [44]. On the positive side, maybe due to their lesser degeneration level, a higher proportion of the OTUs got with Zeale primers were assigned to know taxa (68%, Table 1), providing a deep coverage of lepidopteran prey species.

As both primer sets used in our study amplify regions of the same well-represented COI marker region, the final prey list did not depend of the availability of model species’ sequences in the databases. Moreover, both primers target mini-COI segments of similar size, short enough to be present in faecal samples after digestion [24,30–32]. In fact, the slightly higher amount of primary OTUs yielded by Gillet primers is consistent with the fact that this primer set amplifies moderately shorter fragments than Zeale ones (133 vs 157 bp, respectively). Nevertheless, the more degenerated Gillet primers could have amplified more DNA fragments, generating more OTUs but with a lower assignment to prey taxa whereas Zeale primers produced less OTUs but with a higher assignment to taxa, consistently with their lower degree of degeneration.

The latest molecular study carried out by Galan et al. [31] described the diet of *R*. *ferrumequinum* as mainly consisting in Lepidoptera and Diptera, whereas morphological studies have described Coleoptera and Lepidoptera as the most important prey orders [47,62–64]. In our study Diptera occurs in 96% of the samples, closely followed by Lepidoptera (79%) and Coleoptera (71%). Nevertheless, lepidopterans were the main prey order detected with Zeale primers, followed by dipterans, whereas with Gillet ones coleopterans and dipterans prevailed, lepidopterans falling down to a modest third place. In fact, coleopterans were only amplified by Gillet primers and some of the most important lepidopteran families (namely Geometridae, Noctuidae and Totricidae) were disclosed by Zeale. We report the family Geometridae and frequently occurring species such as *Rhipidia maculata* (Diptera, Limoniidae), *Serica brunnea* (Scarabaeidae) and *Pharmacis fusconebulosa* (Lepidoptera, Hepialidae), for the first time among prey of *R*. *ferrumequinum*.

*R*. *euryale* has been widely considered a moth specialist [62] and, according to Koselj [65] and Dietz [66], lepidopterans make up 90% of its diet. In our study, separate molecular studies performed with the two primers showed a narrow specialization level of *R*. *euryale* for lepidopterans, but seasonally complemented by ephemeropterans, hemipterans, hymenopterans and trichopterans [33,42]. In fact, ephemeropterans had been previously reported as prey of *R*. *euryale* in North Africa [67]. Noteworthy, lepidopterans are almost the only preyed order if using Zeale, whereas Gillet gives similar importance to lepidopterans and dipterans. Four out of the five most frequently occurring lepidopterans—namely *Capsula sparganii* (Noctuidae), *Udea ferrugalis* (Crambidae), *Lycopohotia porphyrea* (Noctuidae), *Scoparia sp*. (Crambidae) where solely amplified by Zeale. The two most preyed species *Capsula sparganii* and *Austrolimnophila ochracea* have not been described before in the diet of *R*. *euryale*.

*R*. *hipposideros* is known to prey mostly upon Diptera Nematocera, followed by Lepidoptera and Neuroptera [68, 69]. In our study, the combined use of both primer sets overall confirms the diet composition depicted in previous studies [42, 63, 67–70], even if the family choices within dipterans and neuropterans differ. When only Gillet primers were used, though, prevalence of dipterans (mainly limonids) inflated, while that of lepidopterans and neuropterans (hemerobids) deflated. For Zeale, instead, dipterans and lepidopterans appeared almost in the same frequencies, closely followed by neuropterans. This results agree with Andreas et al. [47] who reported a highly prevalence of Lepidoptera in the pellets. Some of the most important families within Diptera reported by morphological studies [71], namely Tipulidae, Empididae, Muscidae and Culicidae are also represented within the most frequent prey species in the current study, Empididae only amplified by Gillet and Muscidae only by Zeale. Two of the most frequent limonid prey species—namely *Neolimonia dumetorum* and *Limonia nubeculosa*—had been previously reported by Galan et al. [42]. Conversely, some other frequent limonids such as *Austrolimnophila ochracea*, *Rhipidia maculata*, *Dicranomyia modesta* or along with other frequent prey species—*Hemerobius humulinus* (Neuroptera, Hemerobiidae), *Wesmaelius nervosus* (Neuroptera, Hemerobiidae), or *Pseudatemelia josephinae* (Lepidoptera)—had not been reported before.

Noteworthy, most of the molecular diet studies carried out on bats exclusively with Zeale primers not surprisingly have concluded that moths or/and Diptera were their main prey: e. g. *Barbastella barbastellus*, *Plecotus macrobullaris*, *Chalinolobus gouldii*, *Vespadelus regulus*, *Nyctophilus gouldi*, *Eptesicus nilssonii*, *Myotis brandtii*, *M*. *daubentonii*, *M*. *mystacinus* and *Plecotus auritus* [17, 24, 35, 39]. In some of the studies [35] previously known prey species–such as Coleoptera, Hymenoptera, Isoptera and Trichoptera–were lacking. Notwithstanding the well-settled importance of moths and dipterans as prey of insectivorous bats (e.g. [63,3]), the reliability of trophic scenarios depicted so far only with Zeale primers is still to be ascertained. Therefore, new studies using combination of primers are highly advisable in order to acquire the fullest dietary view whether to confirm the results obtained with Zeale.

Furthermore, the strong primer bias reported herein cast doubts on the results of previous studies comparing the trophic niche overlap between sibling bat species, carried out exclusively with a single primer set (Zeale). For example, a study comparing the niches of *R*. *euryale–R*. *mehelyi* [34] showed a high degree of diet overlap. Razgour et al. [51] obtained similar results for *Plecotus austriacus* and *P*. *auritus*. Some other studies have also analysed the diets of sympatric bat species [40] based on Zeale primers. Even though the diet overlaps these studies reported cannot be denied, other primers may well unveil additional consumed prey and higher levels of resource partitioning among the species pairs.

Last but not least, previous studies have shown that Gillet primers are useful to identify predators’ DNA [42,46]. In this study we identified almost all the faecal samples for their predator, in except from two *R*. *ferrumequinum* samples. Galan et al. [42] argued that a mismatch (T/C) at the 30-end of the reverse primer could be at the origin of their higher rates of amplification failure for some bat species, including *R*. *ferrumequinum*. We also identified DNA remains indicating unexpected interactions, including secondary predation events. Thus, we found one *R*. *euryale* faecal sample containing *Bos taurus* sequences, likely traces of bovine animal excrements coming from the common housefly (*Musca domestica*). *Lichtheimia ramosa* was identified in *R*. *euryale* and *R*. *hipposideros*. This is a fungus living in soil and vegetable wastes that infects both animals and humans. These results must be considered with caution, though, because field contamination cannot be fully discarded.

## Conclusion

On the one hand, the present study shows that the combination of primer sets with different degeneration degrees that amplify different sub-regions of a specific marker allows identifying a broader and more complete prey spectrum for generalist predators like insectivorous bats. The complementarity of the results yielded by both primer sets lie at the species level, since very few prey species’ sequences were amplified by both primers. For instance, thanks to the use of both primer sets we were able to reveal that *R*. *euryale*, though considered a moth specialist, complemented its diet with very diverse prey. On the other hand, our results stress the constraints of the PCR-based metabarcoding diet studies, due to the biases of the many methodological procedures and steps involved in them. Due to biases involving false positives, false negatives and varying affinities to amplify different sequences, we must be extremely cautious when drawing any conclusion from the results gathered, and even when comparing results of different studies. This strong bias when amplifying prey sequences will yield erroneous or at least partial pictures of the trophic requirements of the consumers and resource partitioning among them. In this context, the use of complementary primers improves any assessment of species trophic spectrum and resource partitioning. In this case, we have seen that among the three studied horseshoe bats resource partitioning exists, mostly between *R*. *euryale* and *R*. *ferrumequinum*.

## Supporting information

S1 Supporting Information File Table A. Prey occurrences revealed by each primer set: G, Gillet primers; Z, Zeale primers; Rfe, *R*. *ferrumequinum*; Reu, *R*. *euryale*; Rhi, *R*. *hipposideros*. Table B. Identifications of Zeale OTUs. Table C. Identifications of Gillet OTUs. Table D. Sequences of all the OTUs built with both primer sets.
(XLSX)
